# Neurobiological Promises of the Bitter Diterpene Lactone Andrographolide

**DOI:** 10.1155/2022/3079577

**Published:** 2022-02-01

**Authors:** Rajib Hossain, Cristina Quispe, Jesús Herrera-Bravo, Jorge F. Beltrán, Muhammad Torequl Islam, Shabnum Shaheen, Natália Cruz-Martins, Miquel Martorell, Manoj Kumar, Javad Sharifi-Rad, Fethi Ahmet Ozdemir, William N. Setzer, Mohammed M. Alshehri, Daniela Calina, William C. Cho

**Affiliations:** ^1^Department of Pharmacy, Life Science Faculty, Bangabandhu Sheikh Mujibur Rahman Science and Technology University, Gopalga nj-8100, Bangladesh; ^2^Facultad de Ciencias de la Salud, Universidad Arturo Prat, Avda. Arturo Prat 2120, Iquique 1110939, Chile; ^3^Departamento de Ciencias Básicas, Facultad de Ciencias, Universidad Santo Tomas, Chile; ^4^Center of Molecular Biology and Pharmacogenetics, Scientific and Technological Bioresource Nucleus, Universidad de La Frontera, Temuco 4811230, Chile; ^5^Department of Chemical Engineering, Faculty of Engineering and Sciences, Universidad de La Frontera, Temuco, Chile; ^6^Department of Plant Sciences, LCWU, Lahore 54000, Pakistan; ^7^Faculty of Medicine, University of Porto, Alameda Professor Hernâni Monteiro, 4200-319 Porto, Portugal; ^8^Institute for Research and Innovation in Health (i3S), University of Porto, 4200-135 Porto, Portugal; ^9^Institute of Research and Advanced Training in Health Sciences and Technologies (CESPU), Rua Central de Gandra, 1317, 4585-116 Gandra, PRD, Portugal; ^10^TOXRUN-Toxicology Research Unit, University Institute of Health Sciences, CESPU, CRL, 4585-116 Gandra, Portugal; ^11^Department of Nutrition and Dietetics, Faculty of Pharmacy, And Centre for Healthy Living, University of Concepción, 4070386 Concepción, Chile; ^12^Universidad de Concepción, Unidad de Desarrollo Tecnológico, UDT, Concepción 4070386, Chile; ^13^Chemical and Biochemical Processing Division, ICAR-Central Institute for Research on Cotton Technology, 400019, Mumbai, India; ^14^Facultad de Medicina, Universidad del Azuay, Cuenca, Ecuador; ^15^Department of Molecular Biology and Genetics, Faculty of Science and Art, Bingol University, Bingol 1200, Turkey; ^16^Department of Chemistry, University of Alabama in Huntsville, Huntsville, AL 35899, USA; ^17^Pharmaceutical Care Department, Ministry of National Guard-Health Affairs, Riyadh, Saudi Arabia; ^18^Department of Clinical Pharmacy, University of Medicine and Pharmacy of Craiova, 200349 Craiova, Romania; ^19^Department of Clinical Oncology, Queen Elizabeth Hospital, Kowloon, Hong Kong

## Abstract

Andrographolide (ANDRO), a bitter diterpene lactone found in *Andrographis paniculata* (Burm.f.) Nees, possesses several biological effects such as antioxidant, anti-inflammatory, and organo-protective effects. Scientific reports suggest that it also has neuroprotective capacity in various test systems. The purpose of this review was to synthesize the neuropharmacological properties of ANDRO and highlight the molecular mechanisms of action that highlight these activities. A careful search was done in PubMed and Google Scholar databases using specific keywords. Findings suggest that ANDRO possess neuroprotective, analgesic, and antifatigue effects. Prominent effects were stated on neuro-inflammation, cerebral ischemia, Alzheimer's and Parkinson's diseases, multiple sclerosis, and brain cancer in mice and rats. Furthermore, ANDRO and its derivatives can enhance memory and learning capacity in experimental animals (rats) without causing any toxicity in the brain. Thus, ANDRO may be one of the most promising plant-based psychopharmacological lead compounds for new drug development.

## 1. Introduction

Neurological disorders are very common and important health issues in elderly people [1, 2]. The inflammation plays a crucial role in several neurodegenerative diseases, including Alzheimer's (AD) disease and Parkinson's (PD), multiple sclerosis (MS), and amyotrophic lateral sclerosis (ALS). Oxidative stress is an important factor in the pathophysiology of many chronic diseases such as diseases of the central system or cancers [3, 4]. Therefore, much attention has been given to the discovery and development of neurological drugs from various origins [5, 6].

Current evidence suggests that plants are a promising source of phytochemicals for the treatment of various diseases [7–9], and thus, phytochemicals are considered one of the most popular tools for sound health maintenance throughout the world [10–13]. Diterpenes are evident in their many promising biological activities, including neuroprotective capacity [14, 15].

**Figure 1:**
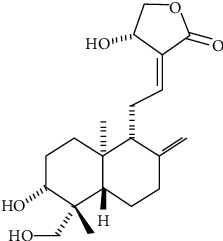
Andrographolide chemical structure.

Andrographolide (ANDRO, C_20_H_30_O_5_; Figure 1), a bicyclic diterpene lactone isolated from *Andrographis paniculata* (Burm.f.) Nees (Family: *Acanthaceae*), has shown diverse biological activities [15–18].

ANDRO has neurobiological properties, such as antidepressant [19], anti-Alzheimer [20, 21], anti-Parkinsonism [22], neuroprotective [23], antioxidant [24], anticancer [25], and cognitive improvement [26] effects. It also shows neuroprotective effects by inhibiting proinflammatory cytokines such as tumor necrosis factor-*α* (TNF-*α*) and interleukin-1*β* (IL-1*β*) production and microglial activation [27, 28]. In addition, ANDRO (0.25–8 mg/kg) is known to exert antifatigue activity in experimental animals [29]. Moreover, solid lipid nanoparticles prepared by ANDRO have been found to improve the transport facility through the BBB in healthy rats [30]. No serious toxic effects in mice were observed with two ANDRO derivatives, 3,19-isopropylidenyl- and 3,19-dipalmitoyl, up to 100 mg/kg [31].

In this sense, the present review is aimed at focusing on ANDRO neurobiological effects.

## 2. Search Strategy

A search was done in the following databases: PubMed/Medline and Google Scholar using the next MeSH terms: “Diterpenes”, “Neuroprotective Agents”, “Andrographolide”, “Animals”, “Central Nervous System/drug effects”, “Diterpenes/pharmacology”, “Diterpenes/therapeutic use”, “Humans”, “Neuroprotective Agents/pharmacology”, and “Neuroprotective Agents/therapeutic use”.

The research was done according to the consensus statement of researchers active in ethnopharmacology and with particular input by the ConSEFS Advisory group [32], and the name of the plant was verified according to the PlantList [33]. No language restrictions were imposed, and manuscripts were evaluated for dose/concentration, administration route, test systems, results, discussion, conclusion, and proposed mechanisms of action.

The following inclusion criteria were considered:

1. Studies developed *in vitro*, *ex vivo,* or *in vivo*, and humans and their derived tissues and cells
2. Studies with ANDRO and its derivatives
3. ANDRO or its derivative joint effects with other chemical compounds
4. Studies with or without proposing mechanisms of action

**Figure 2:**
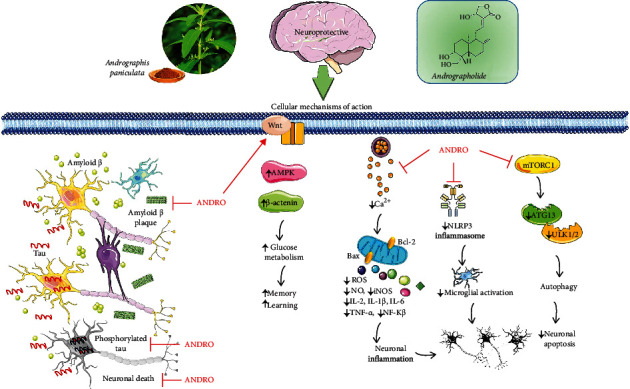
Schematic representation of the most important molecular mechanisms and signaling pathways of andrographolide in central nervous system disorders. ↑: increase; ↓: decrease; ANDRO: andrographolide; ROS: reactive oxidative species; NO: nitric oxide; iNOS: inducible nitric oxide; IL: interleukins; TNF: tumor necrosis alpha; NF-*κ*B: nuclear factor kappa-light-chain-enhancer of activated B cells. ATG 13 autophagy-related protein 13, ULK ½ Unc-51 like autophagy activating kinase.

After careful search and strict analysis, 38 reports (PubMed: 28; Google Scholar: 10) were included. The most important mechanism of ANDRO' effects on AD, PD, MS, cerebral ischemia, intracerebral haemorrhage, neuropathic pain, brain tumour, and depression is summarized in Table 1 and Figure 2.

## 3. The Neurobiological Role of Andrographolide: Molecular Mechanisms and Pathways

### 3.1. Andrographolide and Neurodegenerative Diseases

Neurodegeneration occurs in the CNS and involves the loss of neuronal structure and function, triggered by several factors including CNS inflammation [34, 35]. CNS degeneration involves the progressive chronic loss of neural structure and function, resulting in functional and mental neurological deficiencies [7, 13].

#### 3.1.1. Alzheimer's Disease

Alzheimer's disease (AD) is a chronic neurodegenerative disease, which usually has a slow progression and gradually worsens over time. It is the cause of 60–70% of cases of dementia [36, 37].

Current treatment protocols are not sufficient for effectively preventing AD signs and symptoms [38, 39]. Many natural resources have been discovered in recent decades which can be used as adjuvant therapies in the treatment of AD. [8, 40, 41].

In some recent studies, ANDRO administration (2 and 4 mg/kg i.p.) suppressed the spatial learning and memory function impairment in *Octodon degus* [42], and the proposed mechanisms of ANDRO were as follows: (1) recovery of learning performance and spatial memory, (2) synaptic basal transmission recovery, (3) protection of synaptic proteins, and (4) lowering of amyloid-beta (A*β*) aggregate maturation and phosphorylated tau protein [42].

In another study, ANDRO (2 mg/kg) was found to improve learning/memory by activating Wnt signaling. Wnt is a signaling pathway that enhances glucose metabolism via gene expression and/or activity enhancement of hexokinase, phosphofructokinase, and AMP-activated protein kinase (AMPK) [20, 43].

ANDRO also reduced the A*β* levels and tau phosphorylation and changed amyloid plaques in A*β*PPswe/PS-1 double transgenic male mice [26]. Hence, ANDRO recovered synaptic proteins, increases *β*-catenin levels, reduces active glycogen synthase kinase- (GSK-) 3*β* levels, increases synaptic transmission, and protects long-term potentiation (LTP) at the same time that suppresses postsynaptic-density-protein 95 (PSD-95), GluA2, GluN2B, and Shank decrease in the hippocampus [26]. Besides, the activation of nuclear factor erythroid 2-related factor 2- (Nrf2-) mediated heme oxygenase (HO)-1 expression, ANDRO (1–10 *μ*M) also inhibited A*β*42-overexpression in microglial BV-2 cells [44].

In human microglia cells, ANDRO also inhibited nuclear factor- (NF-) *κ*B translocation via I*κ*B phosphorylation modulation and attenuated A*β*- (1-42-) induced Jun N-terminal kinase- (JNK-) mitogen-activated protein kinase (MAPK) overactivation [45]. ANDRO sulfonate (2.5 and 5 mg/kg, 5 months) was found to inhibit AD *via* mitochondria protection in APPswe/PSEN*Δ*9 double transgenic mice having AD [46].

Other studies narrate that ANDRO treatment during chronic cerebral hypoperfusion suppressed astrocyte activation supported by decreased expression of the glial fibrillary acid protein (GFAP), enhanced brain-derived neurotrophic factor (BDNF) and tyrosine kinase receptor B (TrkB) expression, and reversed upregulated expression of TNF-*α*, IL-1*β*, and caspase-3. Thus, in the rat model of chronic cerebral hypoperfusion, ANDRO improved impaired spatial learning and memory [47].

#### 3.1.2. Parkinson Disease

Parkinson's disease (PD) is described as a gradual loss of midbrain *substantia nigra* dopaminergic neurons [48, 49].

Some researchers have shown that pretreatment with ANDRO (0.5–5 *μ*M) abolished lipopolysaccharide- (LPS-) induced decrease in dopamine (DA) uptake but failed to affect 1-methyl-4-phenyl-pyridine- (MPP-) induced decrease in DA uptake. Thereby, ANDRO (1–5 M) reduced the tyrosine hydroxylase- (TH-) immunoreactive neuron loss and shortened TH-immunoreactive dendrites [22]. Microglia-derived toxic factors include proinflammatory mediators such as reactive oxygen species (ROS), prostaglandin E2 (PGE2), TNF-*α*, inducible nitric oxide synthetase (iNOS), nitric oxide (NO), and cyclooxygenase 2 (COX2) [50, 51].

Anxiety and depressive disorders are frequently comorbid with PD [52]. Thus, a forced swimming test was performed in mice to measure depressive symptoms. After ANDRO (5 mg/kg) treatment, the swimming time was noticeably enhanced showing an improvement of depressive symptoms [53]. Geng et al. [54] provided evidence that ANDRO attenuated DA neuron loss, oxidative stress, and preserved mitochondrial morphology and also reduced mitochondrial malfunctions, reduced cell death, and inhibited GTPase activity in 1-methyl-4-phenyl-1,2,3,6-tetrahydropyridine- (MPTP-) induced PD. Apoptosis is also an important factor that triggers PD.

ANDRO reduced Ca^2+^ influx [55], intracellular ROS production [56], and lipid peroxidation. In addition, ANDRO regulated Bcl-2, Bid, Bax, and apoptosis-inducing factor levels. ANDRO also inhibited the phosphorylation of mitogen-activated protein kinases (p38, extracellular signal-regulated kinase (ERK), and *c*-JNK) [55].

ANDRO (0.1–10 *μ*M) was found to decrease apoptosis and inhibited IL-2 [57] and maybe an antagonist of phorbol-12-myristate-13-acetate (PMA) which stimulated remarkable ROS production [56].

#### 3.1.3. Multiple Sclerosis

Multiple sclerosis (MS) is a well-known immune-mediated disorder, in which insulating covers of nerve cells in the spinal cord and brain are damaged in the CNS [58]. From the MS pathogenesis, it was found that CD4^+^ T-cell-mediated autoimmunity is crucial in MS pathogenesis, mainly for early disease initiation [59, 60]. T-helper type 1 (Th1) cells, characterized by interferon- (IFN-) *γ* production, mediate the MS pathogenesis [61, 62], but IL-17-expressing T-helper cells (Th17) are also involved. CD8^+^, as well as CD4^+^ T cells, was equally immune-stained for IL-17 and IL-17 production inactive areas of MS lesions [63].

ANDRO inhibits the dendritic cells ability and generates peptide-major histocompatibility complexes required for T cell activation. In LPS-treated dendritic cells, ANDRO attenuated the upregulation of the maturation markers I-A^b^, CD40, and CD86 (B7.2) [16]. Besides, ANDRO also suppressed T cell function, IFN-*γ*, and IL-2 production [57]. These effects may contribute to ANDRO's therapeutic potential, ameliorating MS symptoms in autoimmune encephalomyelitis mice through inhibition of T-cell activation and antibody responses directed to the myelin sheath [16].

### 3.2. Andrographolide and Stroke

#### 3.2.1. Ischemic Stroke

Cerebral ischemia occurs when the metabolic demand of brain is not satisfied due to an insufficient blood flow [48, 64]. This involves cerebral hypoxia leading to the death of brain tissues [65, 66]. Cerebral ischemia is one of the serious causes of morbimortality worldwide. The treatment options against cerebral ischemia/stroke are limited [67].

Some studies have shown that ANDRO (0.1 and 1 mg/kg i.p) lowered the infarct volume and neurological deficits and drastically reduced microglia cells in permanent middle cerebral artery occlusion- (pMCAO-) induced rat model [27]. On the other hand, ANDRO showed a neuroprotective effect by reducing inflammation in the MCAO-induced rat model. ANDRO abolished neuroinflammatory markers, such as IL-1*β* and TNF-*α*. ANDRO also suppressed NF-*κ*B activation [27].

Another study showed that ROS production and protein nitrosylation, iNOS, gp91phox/NADPH oxidase 2 (NOX2), IL-1*β*, and HIF-1*α* levels were decreased by ANDRO (5 and 10 *μ*g/kg, i.v.) in cerebral ischemia in rats [68]. Furthermore, NOX2 and iNOS expression were reduced by impairing PI3K/protein kinase B- (AKT-) dependent NF-*κ*B and HIF-1*α* activation in cerebral ischemia in mice [24]. ANDRO, in rat hippocampal cultures, also inhibited GSK-3*β* in a non-ATP-competitive, substrate-competitive way [69].

In a recent study, ANDRO (5–100 *μ*g/kg, i.p.) increased Wnt/*β*-catenin signaling as evidenced by the enhanced nuclear *β*-catenin expression and inhibited GSK-3*β* (pSer9) [70].

#### 3.2.2. Hemorrhagic Stroke

Intracerebral hemorrhage (ICH) occurs within brain tissue or ventricles [71] and is a prime CNS health problem all over the world with high morbimortality rates. So far, no effective strategies exist to treat this disorder, and nearly 20% of patients achieve therapeutic outcomes [72]. There are several causes of, including neuroinflammation and microglial activation. Therefore, prevention and secondary treatment of brain injury are important for patients with ICH [23, 73].

In some studies, ANDRO (1 and 2 mg/kg) was able to reduce neurobehavioral damage, the water content in the brain, alleviate neuronal cell death, and degeneration in ICH-induced SBI rats [23]. Further experimental analysis shows that ANDRO has inhibitory effects on CD11b + and CD16+ microglia cells and attenuated TNF-*α* and IL-6 level by deactivating the NF-*κ*B signaling pathway through reverse phosphorylation of I*κ*B*α* and p65 in ICH rats [23]. Furthermore, in the ICH brain, ANDRO reduced caspase-1-caspase-recruitment domain (ASC) and NLRP3-ASC interaction, thereby inhibiting caspase-1/gasdermin D cleavage and IL-1*β* production [23]. In addition, ANDRO suppresses NF-*κ*B and NLPR3 inflammasome activation via p65 translocation assembly inhibition of NLRP3/ASC/caspase-1 complex [23] and can reduce SBI after ICH [15].

### 3.3. Andrographolide and Neuropathic Pain

Pain sensation occurs when tissue injury is detected by nociceptors [74]. ANDRO (25, 50, 100 mg/kg) is evident to exert analgesic effects by reducing writhing reflex [75]. ANDRO and its derivatives 14-deoxy-11,12-didehydroandrographolide, 14-acetyl-3,19-isopropylidenyl-, and 3,19-dipalmitoyl-derivatives (4 mg/kg) exerted analgesic effects in hot plate and writhing test in mice [31].

ANDRO is also evident to exert analgesic effects in Charles Foster male albino rats [76]. ANDRO (25 mg/20 mL) also attenuated mechanical and thermal hyperalgesia and downregulated the expression of the P2X7 receptor. Besides this, ANDRO decreased TNF-*α* and IL-1*β* expression, increased IL-10 expression, inhibited ERK signaling pathways activation, and also decreased the coexpression of GFAP and P2X7 receptors [77], thus reducing neuropathic pain in the HIV rat model.

### 3.4. Andrographolide and Brain Tumors

Glioma is a tumor occurring in the glial cells of the brain or spine [78] and comprises about 30% of all brain tumors and 80% of all malignant brain tumors in CNS [79]. Targeted therapies for cancer are a rapidly advancing field for treating tumor, and natural products have become the best choice for researchers ([80], Sharifi-Rad et al., 2021b).

ANDRO showed anticancer potential in several cancer cell types [25, 81]. ANDRO (15 *μΜ*) induced cell death of glioblastoma (C6) cells by inducing apoptosis through ROS-extracellular receptor kinase- (ERK-) p53-caspase 7- and PARP-pathways in mouse glioblastoma (C6) cells. ANDRO could increase apoptosis through both phosphorylations of p53 and p53 activation. Because ANDRO increased p53 levels in neural cells [55], findings suggest that ANDRO can promote p53 protein activation, which activates the downstream caspase 7-PARP cascade [82] and is regulated by ERK [83]. In a recent study, ANDRO accelerated RSC96 cell proliferation [84].

### 3.5. Andrographolide and Depression

One of the most common neuropsychiatric disorders in the world is depression [85–87]. Characterized by a variety of signs and symptoms, antidepressants have been increasingly used for depression treatment in daily life, but their multiple side effects and high rates of failure have triggered the researchers' interest to find more effective and safer therapeutic strategies [88]. ANDRO (5 mg/kg) administration can improve depressive-like behaviour, as well as to attenuate the expression of proinflammatory mediators and cytokines, including NO, iNOS, COX-2, IL-1*β*, IL-6 and TNF-*α*, NF-*κ*B signaling (p-p65, p-I*κ*B*α*), and NLRP3 inflammasome assembly (NLRP3, ASC, and caspase-1) in the prefrontal cortex. Besides, ANDRO (5 mg/kg) increased Beclin1 expression and abrogated phosphorylated mTORC1 (p-mTOR), revealing autophagic activity in the prefrontal cortex of chronic unpredictable mild stress mice [54]. Beclin1 stimulates the initial stages of autophagy, and p-mTOR inhibits autophagy through ATG13 and ULK1/2 phosphorylation [89]. ANDRO-generated autophagy can attenuate depressive-like symptoms, inhibits inflammation, and shows antidepressive effects [54].

In another study performed in mice, ANDRO (20 and 50 mg/kg) activated hippocampal BDNF system, thus showing antidepressant effects [19, 90]. Also, ANDRO inhibited the long-term depression in a concentration-dependent way, showing *β*-catenin accumulation and reducing the GSK-3*β* active state [91–93].

## 4. Conclusions

The antioxidant and anti-inflammatory effects of ANDRO and some of its derivatives are well-known. It is also widely recognized that substances with these kinds of properties are cytoprotective and can protect animal organs. This review gives insights on the neuro-pharmacological effects of ANDRO and its derivatives in several test systems. In light of these data, ANDRO can be considered one of the most important neuro-protective phytochemicals that can be considered as an adjuvant treatment in neurodegenerative diseases such as AD, PD, and MS. Further studies are needed to find solutions such as nanocarriers, to increase the bioavailability of ANDRO in order to cross the blood-brain barrier by incorporating in pharmaceutical formulations such as nanoparticles.

## Figures and Tables

**Table 1:** ANDRO neuropharmacological activities and possible mechanism of action.

Neurological/psychiatric disorders	Results/possible mechanisms	References
Alzheimer disease	Neuroprotective ↓ A*β* protein ↓ Caspase-3 expression ↓ Apoptosis ↓ Neuronal cell death	([20], [44], [47], [26], [46])
Parkinson's disease	Neuroprotective ↓ ROS, ↓ NO ↓ TNF-*α* ↓ IL-1*β* ↓ Lipid peroxidation ↓ Apoptosis ↓ Cell death	([22], [94], [95], [52], [50], [51], [55], [56], [57], [54])
Multiple sclerosis	Neuroprotective ↓ T-cell activation ↓ IFN-*γ* ↓ TNF-*α* ↓ IL-1*β*, ↓ IL-2 ↓ Apoptosis	([16], [62], [61], [63], [57], [96])
Cerebral ischemia	Neuroprotective ↓ *β*-Catenin ↓ Caspase-3 ↓ NF-*κ*B ↓ Apoptosis ↓ Neuroinflammation	([24], [27], [68], [69], [97], [70])
Intracerebral hemorrhage	↓ Brain injury ↓ Catenin-1 ↓ TNF-*α* ↓ IL-1*β* ↓ IL-6	([15], [23], [98], [71], [72])
Neuropathic pain	↓ TNF-*α* ↓ IL-1*β* ↓ Neuronal excitability ↓ Central and peripheral pain sensitization	([31], [75], [76], [77])
Brain tumor	↑ p53 and ERK phosphorylation ↑ Caspase-7 ↑ Apoptosis	([83], [81], [82], [84])
Depression	↓ Inflammation ↓ NO, ↓ iNOS ↓ COX-2 ↓ TNF-*α* ↓ IL-1*β*, ↓ IL-6 ↑ *β*-Catenin ↑ BDNF ↑ pSer9	([19], [93], [92], [54], [90], [91], [89])

↑: increase; ↓: decrease; A*β*: amyloid beta; COX-2: cyclooxygenase 2; ERK: extracellular signal-regulated kinase; IFN: interferon; IL: interleukin; NF-*κ*B: nuclear factor *κ*B; NO: nitric oxide; ROS: reactive oxygen species.

## Data Availability

The data used to support the findings of this study are available from the corresponding author upon request.
